# Associate Editor Foreword for *iGIE* International Case Reports Section

**DOI:** 10.1016/j.igie.2023.01.006

**Published:** 2023-03-01

**Authors:** Marvin Ryou, Diogo De Moura

In this volume of *iGIE* we introduce the "International Case Reports" section, which aims to highlight interesting clinical findings and innovative solutions that arise in resource-limited settings. Featured cases may include disease processes that are not commonly seen in tertiary care centers of the Western world and remind the discerning clinician to maintain a broad differential. Additionally, cases may describe novel, thought-provoking solutions that arise when the spirit of ingenuity operates in an environment of restricted means.

Elayat et al^1^ present a case series of using the Ambu disposable bronchoscope (Ambu, Copenhagen, Denmark) as a more economical alternative to a transnasal (ultrathin) gastroscope for difficult nasogastric tube placement in critically ill patients. Rathi et al^2^ describe the use of a snare and spray coagulation mode on an Erbe electrosurgical unit (Erbe, Tübingen, Germany) for treatment of gastric vascular ectasia when argon plasma coagulation is unavailable. These reports underscore the proverb “Necessity is the mother of invention.”
